# Upregulation of miR-128 Mediates Heart Injury by Activating Wnt/β-catenin Signaling Pathway in Heart Failure Mice

**DOI:** 10.1080/15476278.2021.2020018

**Published:** 2021-12-29

**Authors:** Jing-Yao Li, Xin-Chang Li, Yu-Long Tang

**Affiliations:** a Department of Cardiology, Daqing Oilfield General Hospital, Daqing, Heilongjiang Province, China; b Cardiac Intensive Care Center, Daqing Oilfield General Hospital, Daqing, Heilongjiang Province, China

**Keywords:** miR-128, Wnt/β-catenin, heart failure, cardiac hypertrophy, Axin1

## Abstract

Cardiac hypertrophy contributes to heart failure and is pathogenically modulated by a network of signaling cascades including Wnt/β-catenin signaling pathway. miRNAs have been widely demonstrated to regulate gene expression in heart development. miR-128 was routinely found as a brain-enriched gene and has been functionally associated with regulation of cardiac function. However, its role and molecular mechanisms that regulate cardiac hypertrophy remain largely unclear. Adeno-associated virus serotype 9 (AAV9)-mediated constructs with miR-128 or anti-miR-128 were generated and delivered to overexpression or blockade of miR-128 *in vivo* followed by HF induction with isoproterenol (ISO) or transverse aortic constriction (TAC). Cardiac dysfunction and hypertrophy, coupled with involved gene and protein level, were then assessed. Our data found that miR-128, Wnt1, and β-catenin expressions were upregulated in both patients and mice model with HF. Interference with miR-128 reduces Wnt1/β-catenin expression in mouse failing hearts and ameliorates heart dysfunctional properties. We identified miR-128 directly targets to Axin1, an inhibitor of Wnt/β-catenin signaling, and suppresses its inhibition on Wnt1/β-catenin. Our study provides evidence indicating miR-128 as an inducer of HF and cardiac hypertrophy by enhancing Wnt1/β-catenin in an Axin1-dependent nature. We thus suggest miR-128 has potential value in the treatment of heart failure.

## Introduction

Heart failure, one of the most common chronic killers worldwide, is a complex and progressive condition accompanied by reduced cardiac output to meet the metabolic demands of peripheral organs. Heart failure is a clinical syndrome that results from myocardial injury from a variety of causes including infection, hypertension, myocardial infarction, chronic ischemia and hypertrophy. Cardiac hypertrophy is an adaptive response in virtue of an increased physiological stress or mutation, and well characterized with increased left ventricular wall thickness termed as concentric hypertrophy, or dilatation of the left ventricular chamber, termed as eccentric hypertrophy.^1^ This cardiac remodeling serves to compensate for volume-overload or pressure-overload, and pathological hypertrophy is regarded as a predominant cause of cardiac dysfunction and eventually heart failure.^2,3^

Cascades of signaling pathways and a portfolio of genes have been identified to serve as potential targets for therapeutic interventions in the heart disorders.^4–7^ For example, cytosolic calcium homeostasis mediated by sarcoendoplasmic reticulum calcium ATPase (SERCA2a) is a critical regulator in respect to cardiac contraction.^8,9^ Wnt signaling is a well-known evolutionarily conserved and developmental signaling pathway for its role in various respects including inflammation, organ development, tissue homeostasis, embryogenesis, and injury repair.^10,11^ Over the past decade, the critical role of Wnt signaling has been recognized in cardiac physiology and pathophysiology. A canonical Wnt signaling pathway dominantly mediated by a central signal transducer β-catenin has been characterized to play notable roles in activation of heart regenerative process and heart remodeling in response to cardiac injury.^12–14^ Aberrant Wnt/β-catenin activation leads to onset and progression of cardiac dysfunction such as cardiac hypertrophy, fibrosis, arrhythmias, and infarction.^15,16^ Wnt1, a signature element of the early Wnt/β-catenin signaling, is a specific and potent inducer of angiogenesis and fibrosis in heart repair following acute cardiac injury.^17^ However, investigation and detailed understanding of molecular strategy of Wnt1/β-catenin pathway in pathophysiological cardiac hypertrophy and heart failure remain largely elusive.

MicroRNAs (miRNAs) are a class of conserve and small (21–23 nucleotide long) single-stranded noncoding RNAs, which promote cleavage of transcripts and repress translation of target genes through base pairing with their identical or similar sequences in 3ʹUTRs. miRNAs can dynamically modulate diverse cellular process including cell differentiation, development, proliferation, and apoptosis, as well as biological process including angiogenesis, vascular development, myogenesis and calcium handling in response to health, disease, and interventions.^18–21^ MicroRNA-128 (miR-128) was first identified as a brain-enriched miRNA and plays essential roles in the development of nervous system, the maintenance of neural physiological functions, metastasis, and tumorigenesis.^22^ Recent studies have demonstrated profound expression of miR-128 in heart tissues and expanded its role in cardiomyocytes proliferation and heart regeneration.^23,24^ While multiple miRNAs such as miR-18 and miR-223 have been reported as indispensable regulators in cardiac function via regulation of genes related to conductance of electrical signals, cardiovascular angiogenesis, muscle contraction, and morphogenesis,^25–28^ functional correlations of miRNAs with Wnt signaling were controversially reported. For example, miR-210 was addressed to directly represses Wnt Signaling, whereas miR-218 and miR-154 stimulates Wnt signaling by suppressing the Wnt inhibitors DKK2 downstream of cascades.^29–31^ These observations indicate miRNA mediated Wnt/β-catenin strategy is a large complex system involved in a variety of cardiac pathologies, and, to date, the physiological significance of miR-128 in cardiac function and whether Wnt/β-catenin signaling is involved in its regulatory mechanisms underlying progression of heart failure remains largely unknown.

During the past cascades, experimental models have been widely developed for investigation of diverse mechanistic insights into heart failure manipulated with such as thoracic aorta constriction (TAC) surgery, isoproterenol hydrocholoride (ISO), or antiotensin II (Ang II) chronic infusion and myocardial infarction/reperfusion (I/R). In the present study, using animal model of TAC operation and ISO infusion, we specified a novel pathogenic role of miR-128 in development of cardiac hypertrophy and heart failure, by positive modulating Wnt1/β-catenin signaling pathway. This regulatory strategy relies on the direct inhibitory binding of miR-128 to Axin1, a repressor of Wnt1/β-catenin axis. We defined the expression, activation, and potential role of miR-128 and Wnt1/β-catenin signaling components in failing hearts of human patients and mouse model treated by either isoproterenol (ISO) infusion or transverse aortic constriction (TAC) operation. We demonstrate miR-128 and Wnt1/β-catenin are synergistically activated in heart failure, and blockade of miR-128 downregulates Wnt1/β-catenin coupled with reinforced Axin1 expression, leading to ameliorated heart dysfunction. Our study therefore suggests miR-128 and Wnt1/β-catenin as potential cardiac therapeutic targets and provides a promising therapeutic insight into the treatment of cardiac hypertrophy and heart failure.

## Methods

### Human blood samples

All 30 HF patients (Age: 63.4 ± 9.6; 17 male and 13 female) were hospitalized in Cardiac Intensive Care Center, Daqing Oilfield General Hospital and suffered end-stage heart failure. At the same time, 30 healthy subjects (Age: 64.5 ± 11.4; 19 male and 11 female) were recruited as normal control group. The gender, age, and other factors were comparable between groups. The investigation was approved by the Clinical Research Committees of Daqing Oilfield General Hospital and conforms to the principles outlined in the Declaration of Helsinki, and all subjects were provided informed consent. Blood samples were drawn from subjects and plasma was isolated by centrifugation and stored at −80°C until RNA extraction.

### Animal experiments

All C57BL/6 mice (8–10 weeks old, 20–25 g) were purchased from GemPharmatech (Nanjing, China). Mice were bred in a specific pathogen-free condition and maintained under constant temperature and humidity with approval by Daqing Oilfield General Hospital. Age-matched and weight-matched mice were used in this study.

For thoracic aortic constriction (TAC) operation, male C57BL/6 mice were subjected to TAC or sham operation as described previously.^32^ Pressure overload-induced cardiac hypertrophy and cardiac function was assessed by echocardiography two weeks post TAC treatment. For isoproterenol (ISO) infusion, heart failure of mice was chronically induced by continuous infusion with isoproterenol hydrochloride (30 mg/kg/d, Sigma–Aldrich, China) over a period of 14 days with implanted Mini-osmotic pumps (Alzet model 1002 and 1004; DURECT Corp., Cupertino, CA) as described previously.^27^ Negative control mice received same manipulation with saline as control.

### Plasmid construction and in vivo gene transfer

The constructs were generated using the pSilencer™ adeno 1.0-CMV System (Ambion) according to the manufacturer’s instructions and described previously.^32^ Briefly, miR-128 mimic and inhibitor sequence, small hairpin RNA (shRNA) against Axin1 were amplified and cloned into a pcDNA3.1 vector. Recombinant adeno-associated viral vector (rAAV9) was used as a delivery system. The AAV9 vectors with package of the plasmids were freshly prepared in 293 FT cells. Seventy-two hours after transfection, the cells were pelleted through ultracentrifugation and recombinant AAV9 vectors were purified using AAVpro purification kit (Takara, Japan). For *in vivo* gene transfer, the rAAV9-mediated vectors with miR-128 mimics, anti-miR-128, or shAxin1 were administrated into mice via a sublingual vein injection two weeks before ISO infusion or TAC operation. Two- or four-weeks post treatment, the mice were subjected to echocardiography assessment, or euthanized and cardiomyocytes or heart tissues were isolated for subsequent analysis.

### Cardiomyocyte isolation and culture

Cardiomyocytes were isolated from mice using a Neonatal Mouse Cardiomyocyte Isolation Kit (Cellutron Life Technology, Baltimore, MD, USA). The hearts from 1- to 3-d-old C57BL/6 mice were harvested aseptically, weighted, and dissected at sterile. Each heart was digested with stirring at 37°C for 12 min. The homogenates were then spun at 1,200 rpm. for 1 min, and cell pellets were resuspended in D3 buffer and seeded on an uncoated plate at 37°C in a CO_2_ incubator for removing adherent cardiac fibroblasts. After 1 hour of pre-plating, the non-adherent cells were separated and incubated in precoated NS medium supplemented with 10% fetal bovine serum (FBS). After culture for overnight, the NS medium was replaced with serum-free NW medium, and the cardiomyocyte cultures were then ready for experiments 48 h after the initial plating.

### Echocardiography

After TAC surgery or ISO infusion, mice were mildly anesthetized by inhaling 1.0% isoflurane and oxygen at rate of 1 L/min. Cardia function was then examined by Doppler echocardiography with a Visualsonic Vero 2100 System echocardiograph (Visualsonics, CA) using a 18–38 MHz transducer (MS400) as described previously.^13^ Images were standardized to the M-mode short axis view at the LV mid-papillary level. Factors including ejection fraction (EF), fractional shortening (FS), and left ventricular internal diameter (LVID) were determined by calculation as previously described.^33^ Two-dimensional images over a minimum of five consecutive cardiac cycles were recorded per heart.

### Histology staining

The mouse heart tissues were isolated, perfusion-washed with cold phosphate buffered saline (PBS), followed by perfusion-fixed with 4% paraformaldehyde (PFA) for 15 min. The tissues were then subjected to gradient paraffin embedding and sectioned at 5 µm. The section slides were stained with hematoxylin and eosin (H&E) according to standard protocol, and imaged under a photomicroscope (Olympus BH 2, Tokyo, Japan).

### Dual-luciferase reporter assay

According to the online prediction of miR-128 binding sequence in the 3ʹUTR of Axin1 mRNA, the target (WT) sequence and mutant (mut) sequence were verified, synthesized, and constructed into a pGL3-reporter plasmid as described.^24^ The Axin1-luc plasmids were then packaged into rAAV9 expression system and co-transfected with miR-128 mimics or control plasmids into mice as described above. Four weeks after *in vivo* gene transfer, cardiomyocytes were isolated and cell lysates were prepared with a Firefly Luciferase Assay Kit (Beyotime, China) following the manufacturer’s instructions. Lysates were then analyzed for luciferase activity using a Glomax20/20 luminometer (Promega, WI). The relative luciferase value was normalized to the level of renilla luciferase activity in each sample.

### Western-blot analysis

Cells were harvested, washed twice by ice-cold PBS, and subjected to lysis. Proteins (20 µg) from cell lysates were separated by 10% SDS-PAGE and transferred to a polyvinylidene difluoride (PVDF) membrane. The membrane was blocked with 5% skimmed milk and immunoblotted with primary antibodies (rabbit anti-mouse Axin1 (ab55906, Abcam), rabbit anti-mouse Wnt1 (ab85060, Abcam), rabbit anti-mouse β-catenin (ab196204, Abcam)) overnight at 4°C followed by incubation at room temperature with a peroxide-conjugated secondary antibody (Beyotime, China). Proteins were then visualized by an enhanced chemiluminescence method (Beyotime, China) according to the manufacturer’s instructions.

### Quantitative real-time PCR

Total RNA was extracted with TRIzol Reagent (Sigma) following the manufacturer’s instructions. A total 1 µg of RNA was then reverse-transcribed into cDNA using a EasyScript First-Strand cDNA synthesis Supermix (TransGen Biotech, China) following the manufacturer’s instructions. Quantitative real-time PCR (qRT-PCR) was performed using a standard LightCycler 480 SYBR Green I Master protocol and analyzed in triplicate by an LightCycler 96 System (Roche, Switzerland). β-actin or GAPDH was defined as a house keeping gene and used to normalize the relative expression levels of target genes by the 2^−ΔΔCt^ method. Primer used for qRT-PCR were as follows: miR-128 forward 5ʹ-GGTCACAGTGAACCGGTC-3ʹ and reverse 5ʹ-GTGCAGGGTCCGAGGT-3ʹ; Wnt1 forward 5ʹ-GGTGGGGGCATCGTGAACATAG-3ʹ and reverse 5ʹ-GGAGGTGATTGCGAAGATAAACG-3ʹ; β-catenin forward 5ʹ-GCTGACCAAACTGCTAAATGACGA-3ʹ and reverse 5ʹ-TGTAGGGTCCCAGCGGTACAA-3ʹ.

### Statistical analysis

All data were expressed as mean ±SEM and analyzed with GraphPad Prism software version 6.01 (La Jolla, CA). Difference between groups was evaluated for significance with using unpaired two-tailed Student *t* test, or Mann-Whitney test, or one-way analysis of variance (ANOVA). *P* < .05 was considered statistically significant.

## Results

### miR-128 and Wnt1/β-catenin expression is enhanced in hypertrophic and failing hearts

**Figure 1.**
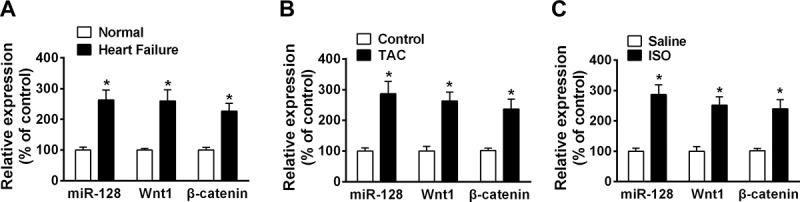
miR-128, Wnt1 and β-catenin mRNA expression are enhanced by heart failure. mRNA was extracted from plasma from heart failure patients (a), hearts tissues from ISO infusion-induced mice model (b) and TAC induced mice model (c) and subjected to quantitative real-time PCR. Each gene expression was normalized by GAPDH and data are presented as means with ±SEM of triplicate samples. n = 30 for a, n = 5 for b and c. *p < .05 compared with control group.

To determine the roles of miRNA in heart failure, we first examined the expression pattern of miR-128 by quantitative real-time PCR in blood samples of human patients with heart failure. We found expression of miR-128 elicited a significant enhanced level in plasma from patients diagnosed with HF, compared to that from healthy control group (Figure 1a). To support this finding, we further established mouse models with HF by either ISO infusion or TAC operation, that caused chronic heart dysfunction and cardiac hypertrophy *in vivo*. Consistent with that in HF patients, miR-128 expression was also remarkably increased in both ISO- and TAC-induced failing mouse heart tissues (Figure 1b-c), suggesting an upregulation of miR-128 in heart failure progression. Wnt1/β-catenin signaling is a conversed pathway and acts as an essential inducer in heart remodeling.^34^ Interestingly, in line with miR-128, both Wnt1 and β-catenin expression showed remarked increase in both HF patient plasma and mouse failing heart, compared to those in the control groups. These findings collectively suggest potential facilitated effects of miR-128 together with Wnt1/β-catenin pathway in the development of heart failure and cardiac hypertrophy.

### TAC operation and ISO infusion drive cardiac hypertrophy and dysfunction

**Figure 2.**
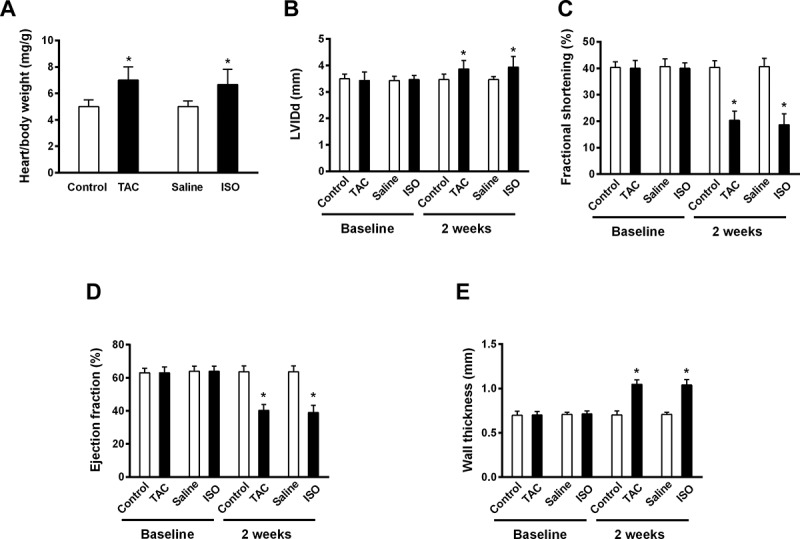
TAC operation and ISO infusion causes cardiac hypertrophic and dysfunctional response in mice. Mice were either treated with or without TAC operation or ISO infusion, and subjected to echocardiographic measurements and hypertrophic analysis at 2-week post treatment. Heart/body weight (a), left ventricular internal diameter (LVID) (b), fractional shortening (c), ejection fraction (d) and wall thickness (e) were analyzed. Data are shown as mean ±SEM. n = 6 for each group. *p < .05 compared with control group.

To assess the pathophysiological effect of TAC operation and ISO infusion on cardiac hypertrophic response and function, echocardiography was performed at pre-treatment and two-week post treatment with either TAC and ISO. Our data clearly demonstrated either TAC-induced pressure-overload injury or ISO continuous treatment resulted in significant cardiac hypertrophic response, as indicated by both higher heart weight and left ventricular internal diameter (LVID), compared to those in the control groups (Figure 2a-b). In addition, significantly reduced fractional shortening and ejection fraction observed in the treated group further confirmed cardiac dysfunction in the mice subjected to TAC operation and ISO infusion (Figure 2c-d), indicating a progressed heart failure under TAC and ISO treatment. Furthermore, higher wall thickness values reflected in TAC- and ISO-treated models reveal an occurrence of concentric hypertrophy and cardiac structural abnormalities following the pressure-overload and ISO-triggered damage (Figure 2e).

### Inhibition of miR-128 suppresses Wnt1/β-catenin expression in response to ISO-induced heart failure

**Figure 3.**
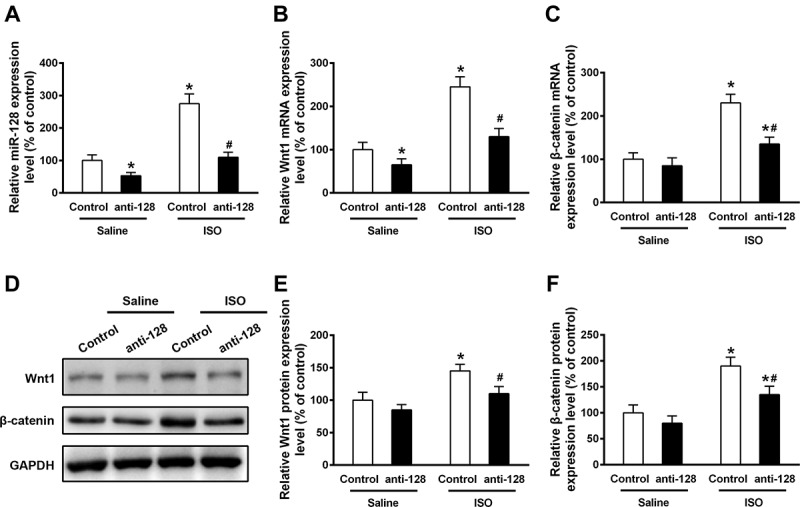
Inhibition of miR-128 impedes the upregulation of Wnt1 and β-catenin induced by ISO infusion in mouse heart. Relative mRNA expression levels of miR-128 (a), Wnt1 (b) and β-catenin (c) were analyzed by qRT-PCR from control or AAV-anti-miR-128 injected mice either untreated or treated with ISO infusion. Western blot was performed to detect the indicated proteins and a representative Western blot is displayed (d). Relative expression of Wnt1 (e) and β-catenin (f) were determined by densitometric analysis, with normalization to internal control GAPDH expression. Data are shown as mean ±SEM. n = 3 for each group. *p < .05 compared with control group treated with saline. #p < .05 compared with control group treated with ISO.

Failing heart models manifested robust Wnt1/β-catenin expression, consistent with miR-128 expression as Figure 1 shown, which prompted us to investigate whether miR-128 may target Wnt1/β-catenin in aggravation of heart failure. To this end, we then examined the effect of miR-128 inhibition on Wnt1 and β-catenin expression in both normal and failing hearts. Anti-miR-128 was cloned into rAAV9 vector and the constructed plasmids were used to perform silencing of miR-128 by *in vivo* gene transduction. C57BL/6 mice were injected with AAV9-anti-miR-128 through a sublingual vein injection 2 weeks before ISO infusion. 2 weeks post ISO treatment, mRNA and proteins were extracted and subjected to qRT-PCR and Western blot analysis to measure the expression of miR-128, Wnt1 and β-catenin. In agreement with the data shown in Figure 1, ISO infusion led to increased mRNA expression of miR-128, Wnt1 and β-catenin (Figure 3a-c) in heart models, compared to control groups. As expected, miR-128 mRNA expression was remarkably inhibited in both control and ISO-induced hearts by silencing of miR-128 (Figure 3a), which interestingly also significantly dampened the up-regulatory effects on Wnt1 and β-catenin in failing hearts induced by ISO treatment (Figure 3b-c). Although inhibition of miR-128 failed to decrease β-catenin expression without treatment of ISO infusion (Figure 3b), that resulted in decrease of Wnt1 expression at the same condition (Figure 3c). A similar decrease of Wnt1 and β-catenin expression by miR-128 inhibition was also observed at protein level (Figure 3d). Western blot analysis showed the protein levels of both Wnt1 and β-catenin induced by miR-128 blockade were lower than the anti-miR-128 untreated group under ISO infusion (Figure 3e-f), but barely showed effect under normal condition (Figure 3e-f). These data together indicated Wnt1 and β-catenin expression were potentiated by heart failure, which was miR-128 activation-dependent.

### Blockade of miR-128 restored cardiac function of mice treated with ISO infusion

**Figure 4.**
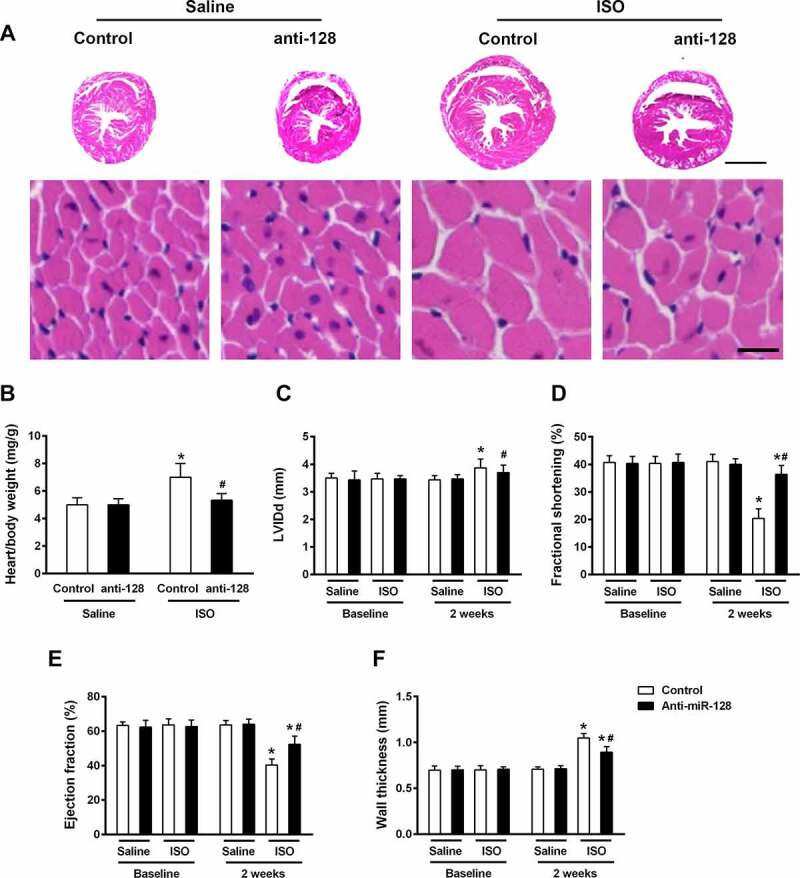
Blockade of miR-128 attenuates cardiac dysfunction induced by ISO infusion. Mice were either injected with control or anti-miR-128 AAV9-constructs, followed by untreated or treated with ISO infusion. (a) HE staining of heart sections from mice was performed and representative images are shown. Scale bar, 2 mm (upper panels), 20 μm (lower panels). Heart/body weight (b), left ventricular internal diameter (LVID) (c), fractional shortening (d), ejection fraction (e) and wall thickness (f) were analyzed by echocardiography. Data are shown as mean ±SEM. n = 6 for each group. *p < .05 compared with control group treated with saline. #p < .05 compared with control group treated with ISO.

We next tested whether miR-128 determine a pathophysiological effect on cardiac hypertrophy and heart failure. Hypertrophic responses and cardiac function were then assessed in hearts from either anti-miR-128 administrated or untreated mice in both control and ISO-induced groups. While miR-128 inhibition hardly caused effect on normal cardiac function, rAAV9-anti-miR128 transduced hearts slices showed moderately preserved cardiomyocytes and reduced hypertrophic responses compared to untreated group in ISO-induced hearts (Figure 4a). Consistently, impaired miR-128 function alleviated hypertrophic responses in respect to reduced heart weight and size and improved cardiac function in contrary (Figure 4b-f), suggesting a vital role of miR-128 silencing in protection against heart failure and cardio hypertrophy progression.

### Axin1 is a target gene of miR-128

**Figure 5.**
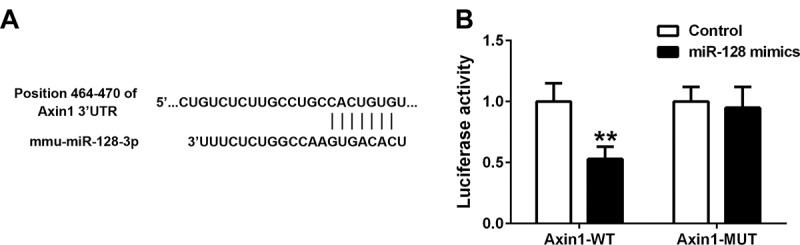
Axin1 is a target gene of miR‐128. (a) The binding sequences of miR-128 and Axin1 3ʹUTR were predicted by the bioinformatics website TargetScan. (b) Axin1-luciferase report plasmids with wt- or mut-sequence were used to co-transfect with either control or miR-128 mimics constructs into mice, and dual‐luciferase reporter assay was performed in isolated cardiomyocytes from each group to detect the luciferase activity. Data are shown as mean ±SEM of three independent experiments. n = 3 for each group. *p < .05 compared with control group.

Axin1 acts as an repressor downstream of Wnt1/β-catenin pathway that regulates β-catenin expression,^35^ which prompted us that miR-128 may play a role in Wnt1/β-catenin cascades via Axin1. To investigate the relationship between miR-128 and Axin1-Wnt1/β-catenin axis, we first identified Axin1 as a binding partner of miR-128 via bioinformatics prediction with online website TargetScan (Figure 5a), suggesting potential direct interaction between miR-128 and Axin1. Moreover, dual luciferase reporter assay indicated that overexpression of miR-128 resulted in a reduced activated response on the Axin1 gene, by contrast to the Axin1-mut gene which was hardly affected (Figure 5b), further confirming Axin1 is a target gene of miR-128 and negatively modulated by miR-128.

### miR-128 promotes Wnt1/β-catenin activation via inhibitory to Axin1

**Figure 6.**
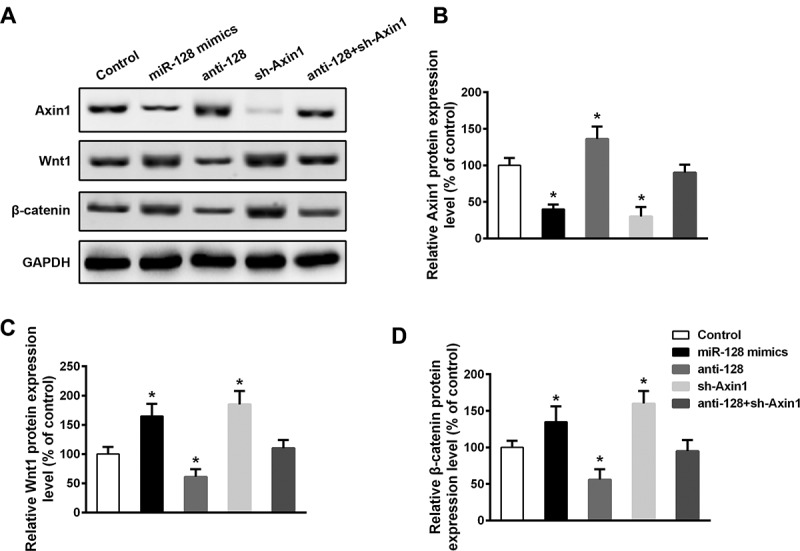
Upregulation of miR‐128 and Axin1 gene silencing promotes activation of the Wnt1/β-catenin signaling pathway in cardiomyocytes. Mice were *in vivo* transfected with miR-128 mimics, or anti-miR-128, or shRNA against Axin1, or anti-miR-128 and shAxin1. Cardiomyocytes were then isolated and subjected to Western blot analysis with indicated antibodies. A representative Western blot is shown (a) and relative protein expression levels of Axin1 (b), Wnt1 (c) and β-catenin (d) were examined by densitometric analysis, normalized to GAPDH. Data are shown as mean ±SEM of three independent experiments. n = 3 for each group. *p < .05 compared with control group.

Given miR-128 plays indispensable roles in regulation of Axin1 and Wnt1/β-catenin, we thereby deepened to align the functional mechanism of the miR-128 on Axin1 activity in the regulation of Wnt1/β-catenin in cardiomyocytes (Figure 6a). Overexpression of miR-128 mimics significantly inhibited Axin1 expression, where by contrast, silencing of miR-128 significantly augmented Axin1 expression (Figure 6b). shRNA targeting to Axin1 was generated to downregulate the Axin1 expression (Figure 6b), which interestingly was unaffected by co-transfection with anti-miR-128. In line, this finding verified miR-128 is required for negative regulation of Axin1 expression. Both Wnt1 and β-catenin expression were shown enhanced by overexpression of miR-128 and decreased by inhibition of miR-128, whereas knockdown of Axin1 had similar effect as overexpression of miR-128 on Wnt1/β-catenin level, indicating opposite role of miR-128 and Axin1 in modulation of Wnt1/β-catenin (Figure 6c-d). While deficiency of both miR-128 and Axin1 failed to cause impact on either Wnt1 or β-catenin expression (Figure 6c-d), a contrary role of miR-128 and Axin1 in Wnt1/β-catenin pathway was thus further confirmed, which also indicated miR-128 promotes Wnt1/β-catenin activation via downregulation of Axin1. Together, these data decisively demonstrated miR-128 mediates the progression of heart failure and the enhancement of Wnt1/β-catenin pathway by downregulatory target on Axin1.

## Discussion

Our study provided the first *in vivo* data to demonstrate that mice with impaired miR-128 expression displayed less susceptible to cardiac hypertrophy and heart failure in response to TAC or ISO treatment. Inhibition of miR-128 attenuates, whereas overexpression of miR-128 drives Wnt1/β-catenin activation coupled with hypertrophic aggravation and cardiac dysfunction, suggesting miR-128 acts as a co-activator of Wnt1/β-catenin in cardiac hypertrophy and heart failure. The synergetic effect of miR-128 on Wnt1/β-catenin activity requires its inhibitory effect on Axin1, a negative regulatory molecule of Wnt1/β-catenin pathway and regarded as a direct target gene of miR-128 by our study. Although miR-128 silencing leads to remarked reduce of Wnt1/β-catenin, co-expression of Axin1 is nevertheless required for the miR-128 modulatory role in Wnt1/β-catenin activation. Collectively, our findings have thus explored a novel pathophysiological role for miR-128 in the stimulation of cardiac hypertrophy and heart failure via direct blemish on the inhibitory activity of Axin1 against Wnt1/β-catenin signaling.

We found that either HF human patients or mouse failing hearts exhibited robustly enhanced miR-128 expression with a higher expression of Wnt1/β-catenin. Not restricted to mouse heart tissues, significant increased expression of miR-128 in human plasma sample, suggesting miR-128 might be used as a circulating biomarker, a diagnostic predictor and a new gene therapeutic target for heart failure. Further, gene therapy through delivery of rAA9-mediated anti-miR-128 gene-alleviated TAC- or ISO-induced hypertrophic and dysfunctional response and declined Wnt1/β-catenin activation, suggesting miR-128 silencing inhibitory to Wnt1/β-catenin is required for protection from the progressive cardiac hypertrophy and heart failure. The importance of miRNA has been well documented for appropriate biological functions including development, differentiation, proliferation, and apoptosis. Dysregulated expression of miRNAs has been identified as a critical cause of aberrant cardiac function in both physiological and pathophysiological conditions. Our finding is consistent with the previous reports that a pool of miRNAs including miR-126, miR214, miR-296, and miR-223 play as a key promoter in cardiac dysfunction via regulation of either cardiovascular angiogenesis or cardiomyocytes apoptosis.^27,32,36^ By contrast, several miRNA genes such as miR-17-92 cluster showed opposite positive function on cardiac function, which govern cardiomyocyte proliferation and heart regeneration after injury, indicating that distinct and balanced roles of miRNAs are required to maintain appropriate regulation in cardiac functions.

As a neuronal-enriched miRNA, miR-128 is associated with central nervous system development and maintenance of neural functions. However, contradictory findings were reported that either downregulation of miR-128 accelerated neuronal proliferation and motor activity by targeting transcription factor E2F3a,^37^ or miR-128 expression protected neurons from apoptosis by suppressing the expression and activation of molecular components downstream of signaling pathways that regulate neuronal excitability such as extracellular signal regulated kinase 2 (ERK) and excitatory amino acid transporter 4 (EAAT4) dominated pathways.^24^ This implies diverse functions and targets of miR-128 underlying molecular mechanisms in the complex network of signaling pathways. These studies, however, provide functional insights of miR-128 into cardiac contexts for us. In our study, miR-128 blockade in mice model was invoked to retard the progression of cardiac hypertrophy and heart failure, which is consistent with a previous study that loss of miR-128 promoted cardiomyocyte proliferation and heart repair by enhancing cyclin-dependent pathways.^23^ Moreover, as a key apoptosis-related element, recent studies have addressed miR-128 inhibition attenuates cardiomyocytes apoptosis and alleviate loss of cardiomyocytes following myocardial ischemia and hypertrophy, by targeting a multitude of ligand-activated transcription factor cascades, including SOX7/IL-33/sST2, PPARG/Akt/Mcl-1, and IRS1/CEBPβ/MAPK7/ signaling.^38–40^ These findings altogether affirm the cardioprotective effect of miR-128 inhibition in the development of heart failure.

Our study provide evidence to point out that miR-128 deficiency impeded the development of cardiac failure and hypertrophy, which concurrently triggered Wnt1/β-catenin downregulation, implying miR-128 promotes cardiac dysfunction through enhancing Wnt1/β-catenin signaling activation. Indeed, miR-128 deficiency caused a significant deduction of Wnt1/β-catenin in both normal and injured cardiomyocytes, whereas overexpression of miR-128 enhanced Wnt1/β-catenin activation in cardiomyocytes without damage, further supporting that miR-128 is required for full activation of Wnt1/β-catenin in cardiomyocytes. TAC operation or ISO continuous infusion are able to induce pressure-overloaded and chronic heart failure, and result in primary heart injury and adaptive dysfunction as manifested by cardiomyocyte hypertrophy, inflammation, cytokine overproduction, and cardiac fibrosis.^34^ Though Wnt1/β-catenin pathway plays canonical roles in embryonic development and tissue homeostatic maintenance,^41^ recent studies have identified Wnt1/β-catenin as a common pathogenic mediator of heart lesions caused by injury, whereas TAC and ISO treatment increased β-catenin expression in line with our finding, and loss of β-catenin attenuated fibrosis and hypertrophy in both cardiomyocytes and cardiac fibroblasts, consequently restoring heart functions.^13,16,34^ Therefore, miR-128 is likely to augment the pathogenic role of Wnt1/β-catenin-dominated axis, that orientates the progress toward heart failure and cardiac hypertrophy after heart injury. Although the underlying physiological causes by miR-128-Wnt1/β-catenin cascade in respect of heart failure were not directly proved, the notions including myocyte hypertrophy, cardiac fibrosis, cardiomyocyte apoptosis, cardiac inflammation and angiogenesis, and myocardial metabolism will be explored in the future work. Our study shows consistent finding with a previous study that demonstrated upregulation of miR-128 promotes cardiovascular calcification through the Wnt signaling pathway.^42^

miR-128 bears a variety of typical motifs, that directly binds and suppresses target genes with identical motifs at 3ʹUTR region. Mechanistically, we defined a negative regulator downstream of Wnt signaling, Axin1, is a direct target gene of miR-128. This was confirmed by that overexpression of miR-128 results in a decline of activity of a luciferase reporter containing the Axin1-3ʹUTR binding sites, while mutation on the Axin1 binding region failed to dampen its luciferase activity in response to miR-128 overexpression. Our data elucidated miR-128 renders Axin1 less capable to inhibit Wnt1/β-catenin pathway. miR-128 deficient cardiomyocytes revealed impaired Wnt1/β-catenin activation, a response contrast to that caused by miR-128 overexpression and knockdown of Axin1. Not surprisingly, downregulation of miR-128 in Axin1 deficient cardiomyocytes had bare effect on reduction of Wnt1/β-catenin, suggesting miR-128 upregulates Wnt1/β-catenin activation and its driven heart failure progression in a Axin1-dependent manner. Similarly, it was reported that miR-128 upregulates EAAT4 expression and cell proliferation in the contexts of neurons by control of Axin1.^24^

## Conclusions

In summary, our findings provided robust evidence for understanding of the molecular action of miR-128 in heart failure and cardiac hypertrophy. Our study identified a novel co-stimulatory function of miR-128 on Wnt1/β-catenin activation, dedicating to the development of cardiac hypertrophy and heart failure. In this context, miR-128 directly targets to Axin1, subsequent inhibiting its negative function on Wnt1/β-catenin signaling pathway. This in turn primes activation of Wnt1/β-catenin and its pathogenic function in heart failure. Our study suggests miR-128 acts potentially as a modern therapeutic target and broadens the therapeutic prospective in treatment of heart failure.
